# MiR-223-3p inhibits angiogenesis and promotes resistance to cetuximab in head and neck squamous cell carcinoma

**DOI:** 10.18632/oncotarget.19170

**Published:** 2017-07-11

**Authors:** Alexandre Bozec, Joséphine Zangari, Mathilde Butori-Pepino, Marius Ilie, Salomé Lalvee, Thierry Juhel, Catherine Butori, Patrick Brest, Paul Hofman, Valérie Vouret-Craviari

**Affiliations:** ^1^ Université Côte d’Azur, INSERM, CNRS, IRCAN, Nice, France; ^2^ Head and Neck University Institute, Nice, France; ^3^ Laboratory of Clinical and Experimental Pathology, Pasteur Hospital, Nice, France; ^4^ Hospital-Related Biobank (BB-0033-00025), Pasteur Hospital, Nice, France; ^5^ FHU OncoAge, Nice, France

**Keywords:** MiRs, tumors, neutrophils, inflammation, anticancer agents

## Abstract

MicroRNAs (miRs) participate in tumor growth and dissemination by regulating expression of various target genes. MiR-223-3p is suspected of being involved in head and neck squamous cell carcinoma (HNSCC) growth although its precise role has not been elucidated. In this study, we showed that miR-223-3p is present in biopsies of HNSCC patients and that its presence is correlated with high neutrophil infiltrate. We found that overexpression of miR-223-3p slightly increased proliferation of the CAL27 squamous carcinoma cell line both *in vitro* and *in vivo*. Moreover, miR-223-3p induced CAL27 apoptosis in an orthotopic xenograft mouse model, counteracting the proliferative effect and resulting in no impact on overall tumor growth. We analyzed the effect of miR-223-3p overexpression on signaling pathways and showed that it induced pERK2, pAKT and AKT, consistent with an increase in cell proliferation. In addition, we found that miR-223-3p reduced the STAT3 level correlating with increased cell apoptosis and inhibited vasculature formation. In HNSCC tissues, miR-223-3p expression was inversely correlated to CD31, highlighting the relationship between miR-223 and vessel formation. Finally, we studied the effect of miR-223-3p on response to selected anticancer agents and showed that cells expressing miR-223-3p are more resistant to drugs, notably cetuximab.

In conclusion, our study is the first to show the antiangiogenic properties of miR-223-3p in HNSCC patients and to question whether expression levels of miR-223-3p can be evaluated as an indicator of eligibility for non-treatment of HNSCC patients with cetuximab.

## INTRODUCTION

There are approximately 600,000 new cases of head and neck squamous cell carcinoma (HNSCC) annually worldwide and HNSCC represents the 6th cause of cancer death [1]. Most HNSCC patients are diagnosed with locally-advanced disease and half of them will die of their disease [2]. Patients with HNSCC presented an altered cytokine profile compared to healthy controls. In particular, IL-8, IL-6, TNF-α, MCP1 and MIP-1α were more expressed in the plasma of HNSCC patients [3, 4]. Neutrophils are important mediators in cancer progression and the neutrophils to lymphocytes ratio is an independent predictor of recurrence in HNSCC [5–7]. Recent studies associated neutrophils with poor clinical outcome in HNSCC patients [8, 9]. Peripheral blood neutrophils from HNSCC patients and healthy donors showed distinct functional differences, among them an increased number of immature stages of neutrophils in HNSCC patients [10]. Several studies demonstrated that a high neutrophil infiltration rate in the tumor was associated with more advanced disease and poor prognosis in HNSCC patients [9, 11]. HNSCC induces recruitment, survival, and release of proinflammatory factors such as CCL4 and IL-8 by neutrophils [11, 12]. Moreover, tumor-infiltrating neutrophils may be a major source of MMP9, which can promote cancer cell invasion and metastasis [12]. Dumitru *et al*. reported that neutrophils released soluble factors which phosphorylated cortactin in HNSC cells and promoted their migration [13]. Furthermore, these authors demonstrated that strong cortactin phosphorylation significantly correlated with strong neutrophilic infiltration in tumor tissues from HNSCC patients [13]. Finally, after neutrophil recruitment in the tumor microenvironment, HNSC cells can modulate the biology of neutrophils, which in turn may facilitate cancer progression [11].

Deregulation of microRNAs, a group of small noncoding RNAs, plays a major role in cancer development [14, 15]. Chen *et al*. recently identified a panel of microRNA deregulations that were observed in HNSCC, including 7 consistently up-regulated microRNAs (miR-21, miR-7, miR-155, miR-130b, miR-223-3p, miR-34b) [15]. Interestingly, miR-223-3p plays a critical role in the maturation and biology of granulocytes [16]. Several studies showed that miR-223-3p was up-regulated in tumor tissue and in plasma of cancer patients, including esophagus and oral cancer patients [17, 18]. Furthermore, it has been shown that platelets and macrophages were able to modulate and promote cancer progression through exosome-mediated delivery of miR-223-3p [19–21]. In this context, miR-223-3p may be one of the main granulocytes-secreted molecular factors able to modulate the biology of cancer cells.

The purpose of this study was to analyze the effects of miR-223-3p on HNSC cells, both *in vitro* and *in vivo*. We examined the impact of miR-223-3p expression on migration, proliferation, apoptosis and drug resistance properties of HNSC cells and on some key molecular signaling pathways implicated in cancer progression.

## RESULTS

### MiR-223-3p expression in HNSCC and neutrophil infiltration

**Figure 1:**
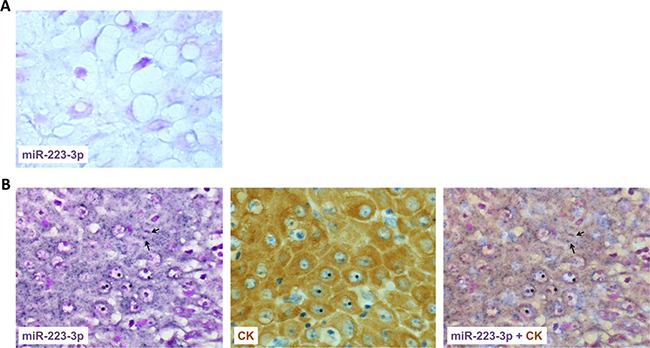
miR-223-3p is overexpressed in head and neck cancer (**A**) miR-223-3p staining of normal epithelium showed few positive dots (asterisks). (**B**) Consecutive sections of T2 head and neck tumor stained for miR-223-3p or pan cytokeratin (CK) showed a high positive signal in CK positive transformed epithelial cells. A representative picture of consecutive sections from 8 tumors stained with miR-223-3p probe and CK is shown (magnification 800×), inset 1600×. Arrows highlighted polymormonuclear cells closed to miR-223-3p positive cells.

MiR-223-3p is highly expressed in the granulocyte lineage and its deregulation is linked to inflammatory and tumor lesions. Whereas some reports indicated that miR-223-3p levels are increased in the serum of HNSCC patients, the expression level of mir-223-3p within tumors of head and neck origin has been poorly studied. In this study, we analyzed the expression of miR-223-3p by *in situ* hybridization in 35 tumors from HNSCC patients. As shown in Figure 1A, low signal was detected in the normal epithelium. On the contrary, T1 and T2 HNSCC exhibited a high expression level of miR-223-3p in CK-positive epithelial cells (Figure 1B). This expression decreased with the size of the tumor (Table 1). Neutrophils being a major source of miR-223-3p, we aimed to characterize the presence of these cells in HNSCC. As shown in Table 1, and illustrated in Figure 1B, some neutrophils are closed to miR-223-3p positive cells. As illustrated in Table 1, we observed a correlation between high neutrophil infiltration and high miR-223-3p expression levels.

**Table 1:** Association of miR-223-3p levels with neutrophil infiltrate and CD31 expression

	Neutrophil		*P*-value	MiR-223-3p staining			*P*-value	CD31 expression			*P*-value
	Low	Medium + High		Low	Medium + High	% high exp		Negative	Positive	% high exp	
Control	6	2	0.042*	6	2		0.05*	8	0		0.03*
Early-stage T	2	20	0.001^#^	2	20	90	0.001^#^	17	5	21	0.2^#^
Advanced-stage T	9	4	0.999 ±	10	3	23	0.99 ±	3	10	77	0.001 ±

Statistical analysis: Fisher's exact test. *Control vs. Tumors. ^#^Control vs. Early-stage Tumors. ± Control vs. Advanced-stage Tumors.

Quantification of neutrophil infiltrate, miR-223-3p staining and CD31 expression from a control group made of 8 healthy people, a group made of 22 HNSCC patients with early-stage (T1 or T2) tumors and a group made of 13 patients with advanced-stage (T3 and T4) tumors was performed by a trained pathologist as following. A sample was considered to display low expression if the percentage of positive cells was from 0 to 10%, median expression (med) if the percentage is between 10 to 50% and high expression if more than 50% of the cells were positive. Negative scoring for CD31 staining corresponds to less than 30% positive cells. Three random fields per tumor were analyzed. This contingency table highlights a correlation between the presence of neutrophils and the expression of miR-223-3p and an inverse correlation between the presence of miR-223-3p and the expression of CD31.

### Effect of miR-223-3p on *in vitro* cell proliferation and migration

**Figure 2:**
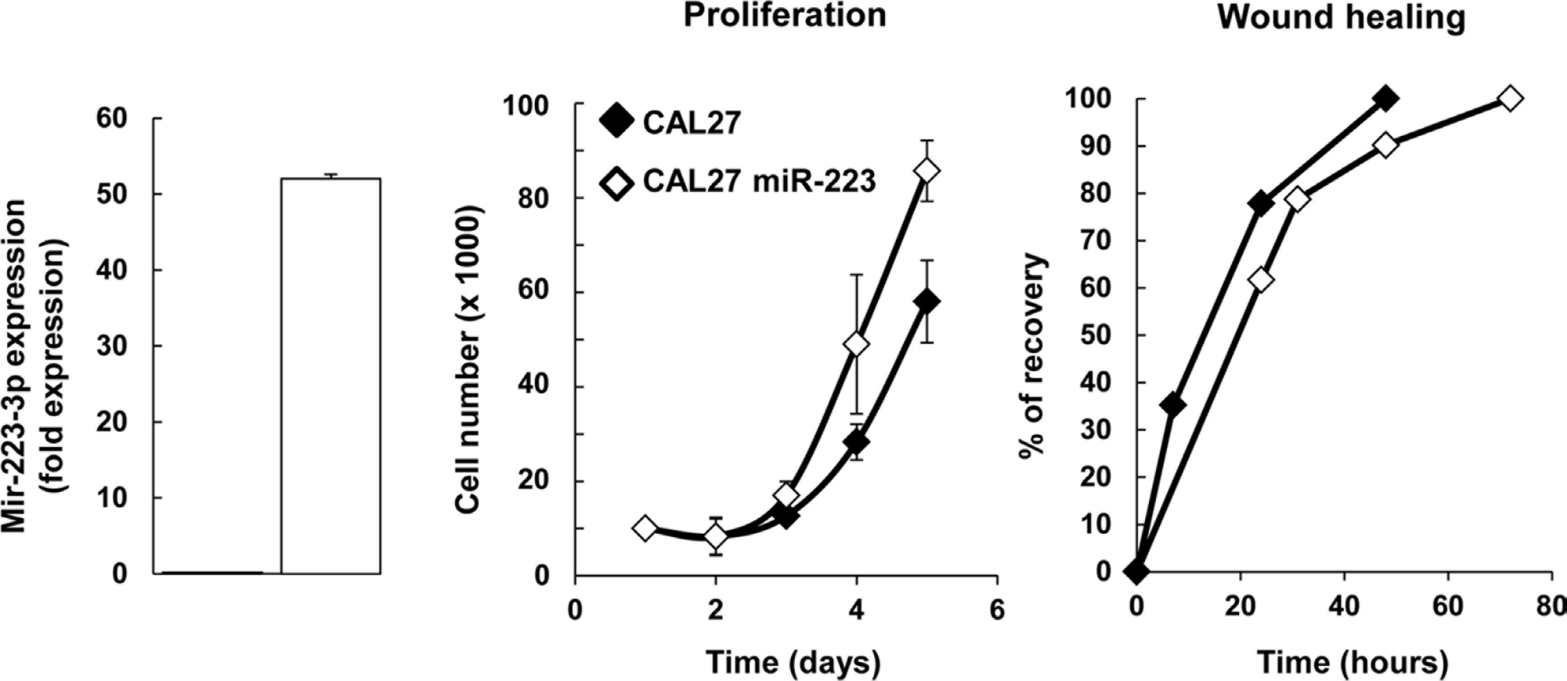
miR-223-3p induced CAL27 proliferation (**A**) Characterization of CAL27 miR-223-3p cells. Total RNA was isolated from CAL27-Luci (named CAL27) and CAL27-Luci miR-223-3p (named CAL27 miR-223-3p) cells and RT-PCR analysis was performed. We showed that miR223 is overexpressed in transfected cells. Rnu19 RNA was used as internal control; Cp value for CAL27 cells was 32.60. (**B**, **C**) Expression of miR-223-3p in CAL27 cells improved cell proliferation (*n* = 4), whereas it had no effect on cell migration. (C) Confluent monolayers were scratched with a yellow tip and cell migration was expressed as the percentage of wound recovery (*n* = 3). Data are presented as mean ± sem.

Since HNSCC expresses high levels of miR-223-3p, we aimed to characterize its effect on cell proliferation, migration and survival. First, we engineered a head and neck cancer cell line overexpressing miR-223-3p by transducing CAL27 cells with hsa-miR-223 and luciferase plasmids. RT-qPCR analysis of total RNA isolated from CAL27 and CAL27 miR-223 cells confirmed that miR-223-3p was overexpressed in transfected cells (Figure 2A). It is known that MiRs modulate the transcription of their target genes. Recently it was shown that miR-223 decreased activation of EGF receptor [22]. Because EGFR plays crucial roles in the biology of head and neck cancer cell lines, we verified that miR-223-3p did not inhibit the EGFR transcription/translation program (Supplementary Figure 1). Further, we analyzed the effect of miR-223-3p on CAL27 cell proliferation and showed that CAL27 miR-223 cells displayed increased cell proliferation as compared to control cells (Figure 2B). However, expression of miR-223-3p in CAL27 cells had no effect on cell migration, as illustrated in the wound-healing assay (Figure 2C).

### Effect of miR-223-3p on *in vivo* tumor growth

**Figure 3:**
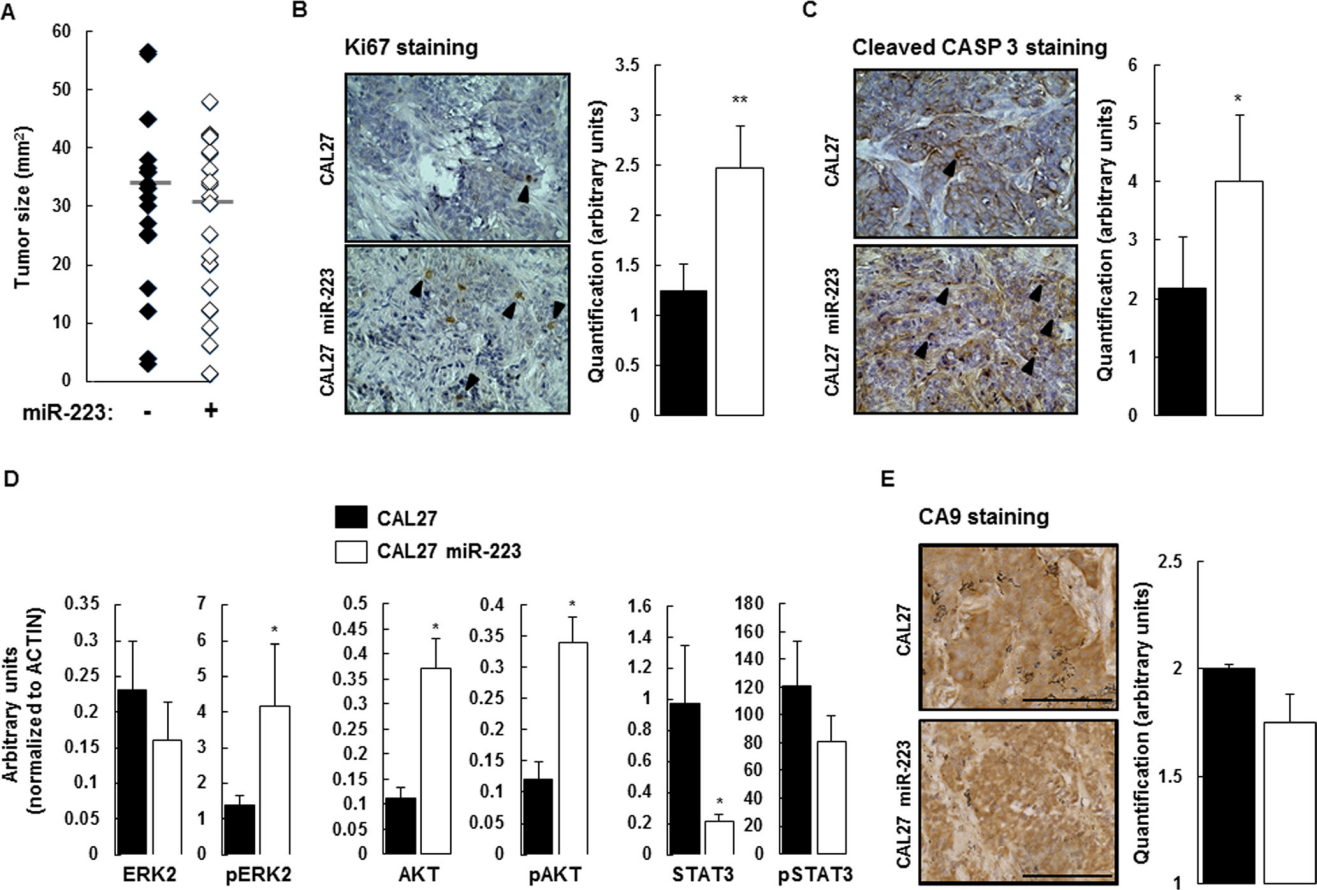
Effect of miR-223-3p on tumor biology (**A**) At day 14, the mice were sacrificed and the tumors were extracted for measurement. Despite the effect of miR-223-3p on cell proliferation, we observed no significant difference between the two experimental groups (*n* = 17). (**B**) Cell proliferation (evaluated by Ki67 staining) within tumors from mice injected with CAL27 miR-223-3p increased. Representative results are shown in the panels on the left and the number of proliferating cells (nuclear Ki67 staining) per tumor was determined as indicated in the Materials and Methods section. (**C**) Similarly, the number of apoptotic cells (evaluated by Cleaved Caspase-3 staining) increased in tumors from mice injected with CAL27 miR-223-3p. Representative pictures are shown on the left. Quantification of the number of apoptotic cells, on the right. (**D**) Immunoblot analysis of ERK2, AKT, STAT3 and their active phosphorylated forms in tumors from CAL27 and CAL27 miR-223-3p mice. Results showed that STAT3 protein is downregulated in cells that express miR-223-3p. (**E**) The hypoxic status of the tumors from both CAL27 and CAL27 miR-223-3p injected mice is comparable. CA9 staining was used as a read-out of hypoxia. Representative pictures are shown on the left. Quantification of the number of hypoxic cells, on the right. Data, shown as arbitrary units, are representative of 5 mice per group (mean ± sem).*P ≤ 0.05. Bar = 100 μm.

We used an orthotopic xenograft model consisting of implantation of CAL27 and CAL27-miR-223 cells in the mouth floor of nude mice to characterize the effect of miR-223-3p on tumor implantation and tumor growth. One day after the injection, we analyzed luciferase activity and kept positive mice for the study, as illustrated in Supplementary Figure 2A and 2B. The mice body-weight follow-up did not demonstrate significant differences between the 2 experimental groups of mice (Supplementary Figure 2C). At the end of the experiment, tumors were collected and measured. No significant difference was found, as illustrated in Figure 3A.

Knowing that miR-223-3p slightly, but consistently, increases CAL27 proliferation *in vitro*, we aimed to characterize its effect *ex vivo*. Cell proliferation was evaluated by Ki67 immunostaining on tumor tissue sections collected from mice receiving CAL27 or CAL27 miR-223 cells. As expected, CAL27 cells were positive for Ki67 staining. We observed that overexpression of miR-223-3p more than doubled Ki67 expression as compared with CAL27 mock cells (Figure 3B). To reconcile this result with our observation that miR-223-3p increases neither the tumor engraftment nor tumor size, we analyzed the effect of miR-223-3p on cell apoptosis. Cleaved caspase 3 staining was used to measure the apoptotic index of tumor cells. Within CAL27 tumors, some cells were stained with anti-cleaved caspase 3 antibody. We observed that CAL27 miR-223 tumors exhibited a higher apoptotic index, as compared with CAL27 tumors (Figure 3C). Thus, miR-223-3p-increased proliferation was counterbalanced by increased apoptosis. This observation could explain why tumors from mice receiving CAL27 miR-223 cells were the same size as mice receiving control cells.

Furthermore, we studied the activity of signaling pathways known to be involved in cell proliferation and cell death, namely ERK2, AKT and STAT3. Total protein extracts from CAL27 and CAL27 miR-223 tumors were analyzed by western blot detecting both total and active phosphorylated forms of ERK2, AKT and STAT3 antibodies (Supplementary Figure 3). Quantification of the results, shown in Figure 3D, indicated that miR-223-3p induced up-regulation of AKT expression and down-regulation of STAT3 expression. A significant increase in pERK2 and pAKT activity was also observed in CAL27 miR-223-3p tumors as compared with CAL27 tumors.

Growing tumors are hypoxic, and miR-223-3p has been reported to attenuate hypoxia-induced vascular remodeling of pulmonary cells [23]. We used carbonic anhydrase 9 (CA9) staining as a read-out of hypoxia and observed that the hypoxic status of CAL27 and CAL27 miR-223 tumors was comparable. Indeed, the 2 experimental groups showed high levels of hypoxia within tumors, as indicated by strong nuclear and cytoplasmic CA9 staining. Diffusion of CA9 within the cytoplasm revealed that hypoxia occurred, but at an undetermined time (Figure 3E). Finally, histological analysis of tumor sections stained with hematoxylin and eosin did not reveal necrosis (Supplementary Figure 3B).

### MiR-223-3p decreases tumor neo-angiogenesis *in vivo*

**Figure 4:**
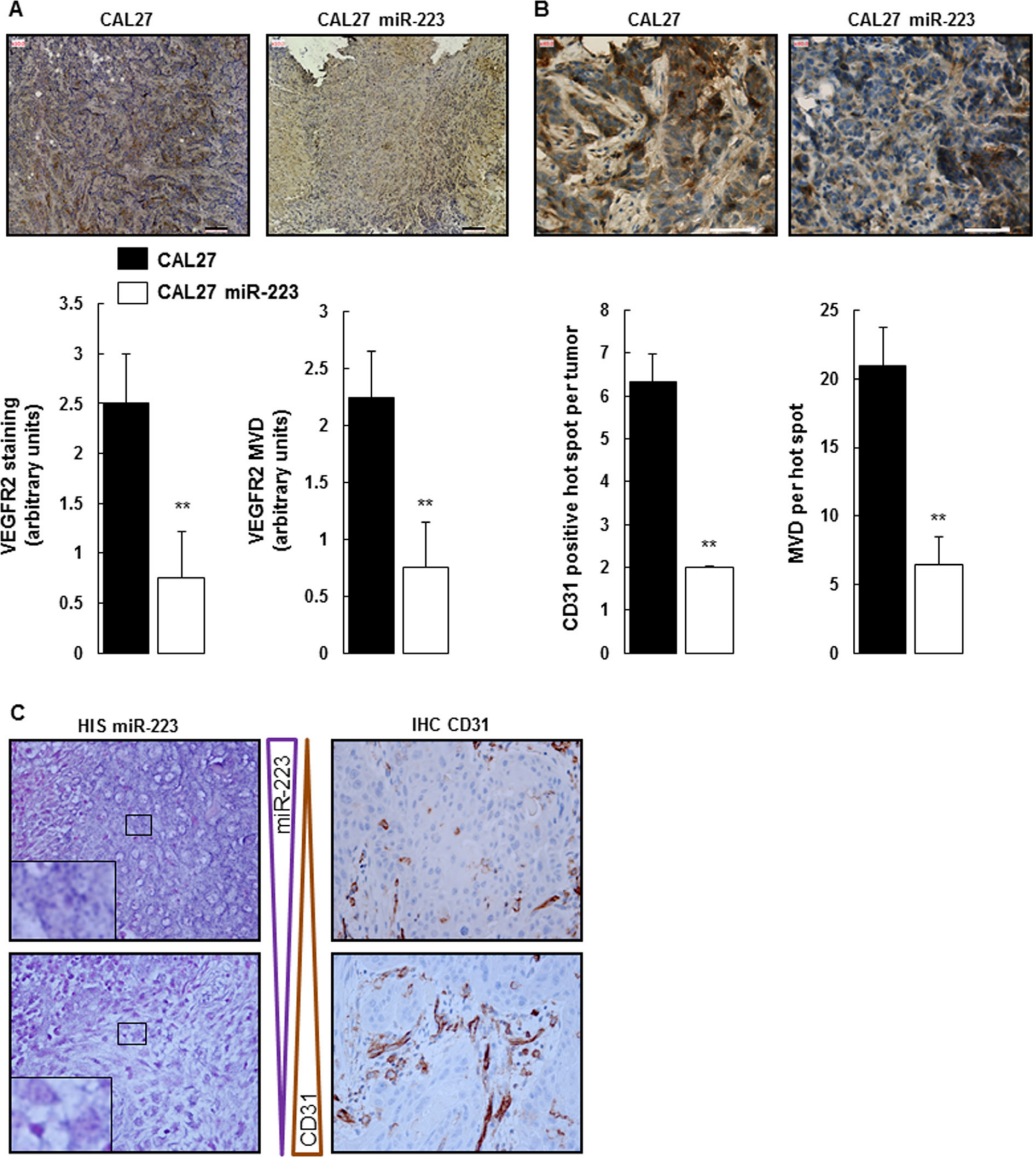
miR-223-3p inhibited neoangiogenesis (**A**) Tumors from mice injected with CAL27 miR-223-3p showed less VEGFR2 staining and MVD. Representative pictures of VEGFR2-stained MVD are shown on the left. Quantification of both VEGFR2 staining and MVD on the right. Bar = 100 μm. Data, shown as arbitrary units, are representative of 5 mice per group (mean ± sem). **P ≤ 0.01. (**B**) Neo-angiogenesis characterized by CD31 staining. Representative pictures of CD31 staining are shown on the left, quantification of the number of CD31 positive hot spots per tumor and the number of MVD per hot spot on the right. Bar = 50 μm. Data are representative of 5 mice per group (mean ± sem).**P ≤ 0.01. (**C**) CD31 staining is inversely proportional to miR-223-3p staining in human head and neck cancer. Pictures are representative of 35 tumors. Magnification 800×.

STAT3 was down-regulated in CAL27 miR-223 tumors. Since STAT3 expression is linked to neo-angiogenesis, we characterized more fully the effect of miR-223-3p on tumor vasculature. To do so, we first analyzed the expression of VEGFR2 by immunostaining. As shown in Figure 4A, CAL27 tumors expressed a high level of VEGFR2. This expression level significantly decreased in CAL27 miR-223 tumors. Furthermore, we quantified micro-vessel density (MVD) based on VEGFR2 staining and confirmed that the expression of miR-223-3p correlated with less neo-angiogenesis. To confirm this interesting result, we performed an independent assay, based on CD31 staining and quantification of the MVD hot spot (Figure 4B). Both approaches confirmed that miR-223-3p decreased neo-angiogenesis.

Importantly, this result has been confirmed on tumor tissue from HNSCC patients. As shown in Figure 4C and Supplementary Figure 4A, areas of high miR-223-3p staining correlated with section of low CD31 expression, whereas areas of low miR-223-3p staining were associated with high CD31 expression. MVD hot spots were quantified (Supplementary Figure 4B), and the result confirmed what was shown on Figure 4C, with a 3.6-fold higher miR-223-3p presence in biopsies with low CD31 staining levels. Finally, quantification of neutrophil infiltrate, miR-223-3p staining and CD31 expression from a control group made of 8 healthy people, a group made of 22 HNSCC patients with early-stage (T1 or T2) tumors and a group made of 13 patients with advanced-stage (T3 and T4) tumors highlighted the correlation existing between these 3 indicators. As expected, healthy people showed low neutrophil infiltrate, low miR-223-3p staining and low CD31 expression. On the contrary early-stage tumors were characterized by high neutrophil infiltrate, high miR-223-3p staining and low CD31 expression. This result first suggested a correlation between the presence of neutrophils and miR-223-3p and second indicated that the presence of miR-223-3p dampened the expression of CD31. Advanced tumors (T3 and T4) demonstrated larger numbers of CD31 positive vessels and weaker levels of miR-223-3p. These two later events are correlated with less miR-223-3p staining, highlighting once again the correlation between the presence of neutrophils and the presence of miR-223-3p in tumor cells. Finally, comparing miR-223-3p presence and CD31 expression in the group with early-stage tumors, we observed that 90% of the patients expressed high levels of miR-223-3p whereas 21% expressed high levels of CD31. The inverse correlation was observed in the group with advanced-stage tumors with 23% of the patients being positive with the anti-miR-223-3p probe and 77% being high CD31 expressers. In conclusion, we showed here that the presence of miR-223-3p correlated with a decrease in tumor neo-angiogenesis.

### MiR-223-3p increases tumor resistance to anticancer agents

**Figure 5:**
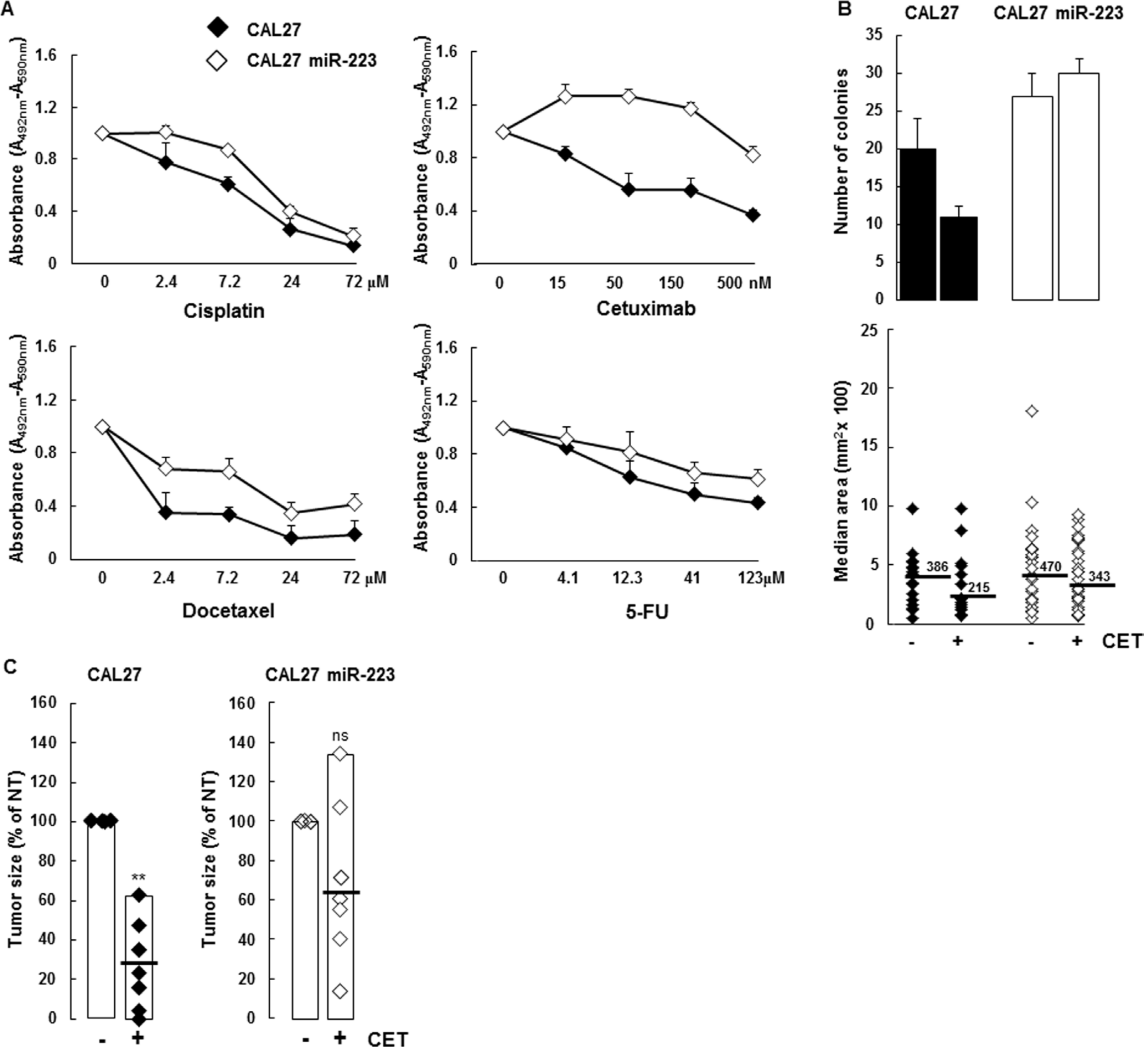
miR-223-3p reversed cetuximab cytotoxic effect (**A**) CAL27 and CAL27 miR-223-3p cells were treated with the indicated doses of cisplatin, cetuximab, docetaxel and 5-FU for 48 hours and cell proliferation and viability were measured. (**B**) CAL27 cells expressing the miR-223-3p are resistant to cetuximab. Cells were treated with cetuximab (50 nM, respectively) and the number of clones was determined as indicated in the Materials and Methods section. Representative pictures are shown on the left, quantification of both the surface and the number of the colony on the right. Data are representative of 2 independent experiments. (**C**) Mir-223-3p-expressing tumors are more resistant to cetuximab. At day 14, the mice were sacrificed and tumors were extracted for measurement. Data are expressed as mean ± sem. *P ≤ 0.01 (*n* = 10).

MiR-223-3p expression is reported to modulate chemoresistance. Given that anticancer agents are used routinely to treat HNSCC, we analyzed the impact of miR-223-3p expression on cisplatin, docetaxel, 5-fluorouracil and cetuximab treatment in a survival assay. As shown in Figure 5A, more XTT incorporation was observed in CAL27 miR-223 cells versus control CAL27 cells. In particular, we observed that CAL27 miR-223 cells were less sensitive to cisplatin, docetaxel, and 5-fluorouracil than CAL27 cells. This drug-resistance effect was particularly evident for the anti-EGFR monoclonal antibody cetuximab. This result indicates that expression of miR-223-3p increases resistance to anticancer agents. To confirm this observation, we performed an independent assay, based on clonogenic growth, in which we quantified the number of colonies and the size of each colony. As shown in Figure 5B, we confirmed that miR-223-3p expression increased resistance to anticancer agents. In particular, miR-223-3p cells were more resistant to cetuximab treatment with a twofold increase in colonies as well as colonies with a higher median area (343 versus 215 mm^2^), as illustrated in the lower panel and in Supplementary Figure 5.

Based on this *in vitro* result, we tested the effect of cetuximab *in vivo*. As expected, cetuximab treatment inhibited tumor growth by 70% in mice injected with CAL27 cells (Figure 5C). In contrast, cetuximab treatment did not efficiently decrease CAL27 miR-223 tumor growth, confirming that miR-223-3p promoted resistance to cetuximab.

## DISCUSSION

Small non-coding RNAs, referred to as Micro-RNAs or miRs, are known to regulate the expression of target genes at the post transcriptional level. As they are involved in various biological processes, such as cell proliferation, metabolism and differentiation as well as cell death, they are suspected of actively participating in tumor growth and propagation [24]. Over the past decade, many studies using microarray profiling and qRT-PCR analyses aimed to identify miRs differentially expressed between malignant HNSCC versus normal tissues. Despite never having been analyzed in depth, miR-223 has been consistently described as overexpressed in HNSCC [15]. We focused our attention therefore on this particular miR, and showed that (i) miR-223-3p was overexpressed in T1 and T2 HNSCC patients, (ii) miR-223-3p slightly increased proliferation of the human CAL27 head and neck cancer cell line *in vitro*, (iii) miR-223-3p did not impact CAL27 orthotopic tumor xenograft growth, and (iv) miR-223-3p decreased tumor neo-angiogenesis and increased tumor resistance to anticancer agents.

MiR-223-3p expression is principally found in bone marrow with preferential expression in the myeloid lineage [25]. The provenance of miR-223-3p in tumor cells remains an open question. At least two hypotheses could be envisaged. First, miR-223-3p, which is localized on the X chromosome, could be on, or close to, a fragile site. These fragile sites are known to predispose to DNA instability and could be amplified during the process of cell transformation, thus leading to miR-223-3p over-expression. Such a process has been described for many miRs [26]. Second, miR-223-3p is produced by neutrophils and shuttles to the tumor cells via exosomes [27]. In agreement with this hypothesis, we observed considerable neutrophil infiltration in HNSCC tissue and a correspondence between areas with strong neutrophil infiltration and areas with high miR-223-3p expression (Figure 1 and Table 1). Globally, both neutrophil infiltration and strong miR-223-3p expression in tumor cells were associated with T1 and T2 tumors (Table 1). However, this correlation has not been observed with higher tumor size (T3 and T4), a discrepancy that can be explained by the few cases included in our cohort. In a recent study, Liang *et al*. demonstrated that miR-223-3p delivered by platelet-derived microvesicles promoted lung cancer cell invasion via targeting tumor suppressor EPB41L3 [21]. Of interest, high expression levels of miR-223-3p, in association with high miR-155-5p and low miR-126-3p, constitute a plasma signature significantly associated with a higher risk for progression in adenocarcinoma patients [28]. However, the role of miR-223-3p in cancer progression is controversial and some studies found that miR-223 inhibited cancer cell invasion and migration [29, 30]. Thus, in the present study, we decided to explore the exact impact of miR-223-3p on HNSCC cell biology, both *in vitro* and *in vivo*.

*In vitro*, we found that miR-223-3p triggered a moderate increase in tumor cell proliferation but did not modify cell migration. Contradictory results have been reported in the literature regarding the impact of miR-223-3p on tumor cell proliferation and migration [29–33]. Yang *et al*., for instance, showed that miR-223-3p negatively regulated the growth and migration of nasopharyngeal carcinoma cells by reducing expression of the transcription factor of Maf family members MAFB [29]. In contrast, Zhang *et al*. found that miR-223-3p functioned as an oncogene in human colorectal cancer cells and demonstrated that reducing miR-223-3p expression resulted in decreased cell proliferation, migration and invasion [32]. This conflicting evidence indicates that, depending on the cellular origin of the tumor as well as the nature of the tumor cell transformation, miR-223-3p could either induce tumor suppression or promote tumor growth.

To characterize the effect of miR-223-3p more precisely, we used an orthotopic xenograft model but did not observe a significant difference between CAL27 and CAL27 miR-223 tumors. Indeed, the higher proliferation index in CAL27 miR-223 tumors as compared to CAL27 tumors was counterbalanced by the higher apoptotic index, thus resulting in comparable tumor sizes. The pro-apoptotic effect of miR-223-3p has already been demonstrated in various experimental conditions and on some types of cells such as endothelial cells, hepatocytes and osteoblasts [34–37]. Immunoblot analysis of CAL27 miR-223 tumors showed increased pERK2, AKT and pAKT expression as compared with CAL27 tumors. Stimulation of the ERK-AKT pathway in CAL27 miR-223 tumors could explain the increased proliferation index found in these tumors. In a recent study analyzing microRNA expression deregulation in oral squamous cell carcinoma, Manikandan *et al*. identified 5 up-regulated microRNAs including miR-223-3p, which was associated with advanced tumor stage [18]. Moreover, similarly to our study, the authors found that microRNA deregulation in oral squamous cell carcinoma resulted in activation of PI3K/AKT signaling pathway genes.

We observed that CAL27 miR-223 tumors exhibited a significant down-regulation of STAT3 expression as compared with CAL27 tumors. This result is not surprising since STAT3 is acknowledged to be one of the miR-223-3p direct target genes. For example, Chen *et al*. demonstrated that miR-223-3p regulated TLR-triggered IL-6 and IL-1β production in macrophages by targeting STAT3 [38]. Such finding concords with our results showing that CAL27 miR-223 tumors, characterized by a low STAT3 expression level, exhibited markedly reduced tumor angiogenesis as compared with CAL27 tumors (Figure 5). Similarly, STAT3 decoy oligonucleotide has been reported to decrease proliferation, migration and tubule formation of endothelial cells *in vitro* and to inhibit tumor angiogenesis in murine HNSCC xenografts [39]. This evidence indicates that miR-223-3p exhibits an antiangiogenic effect and that this effect may be attributed, at least in part, to the miR-223-3p-induced down-regulation of STAT3. The antiangiogenic effect of miR-223-3p is corroborated by observations made on tumor tissue from HNSCC patients, thus indicating that areas of high miR-223-3pexpression displayed low CD31 IHC staining, and vice versa (Figure 4C and Table 1).

This antiangiogenic effect of miR-223-3p has already been reported by several authors [34, 40]. Shi *et al*. showed that miR-223-3p was an antiangiogenic microRNA that prevented endothelial cell proliferation, at least partially, by targeting β1 integrin [40]. In another recent study, Liu *et al*. demonstrated that administration of antagomir-223-3p promoted angiogenesis in rats with spinal cord injury [34]. However, there are few data on the role of microRNAs and, in particular, miR-223-3p, in tumor angiogenesis. Mathsyaraja *et al*. showed that the expression of miR-21, miR-29a, miR-142-3p and miR-223-3p increased in myeloid cells during tumor progression in mouse models of breast cancer and melanoma metastasis. Furthermore, they demonstrated that a loss-of-function approach using selective depletion of the miR-processing enzyme Dicer in mature myeloid cells blocks angiogenesis and metastatic tumor growth [41]. To our knowledge, the present study is the first to demonstrate the antiangiogenic properties of miR-223-3p in patients with HNSCC.

Hence, we examined the impact of miR-223-3p on tumor resistance to anticancer agents. *In vitro*, we found that CAL27 miR-223 cells were less sensitive than CAL27 cells to conventional chemotherapeutic agents commonly used in HNSCC (namely cisplatin, 5-fluorouracil and docetaxel), as well as to the anti-EGFR monoclonal antibody cetuximab. As the drug resistance effect induced by miR-223-3p was most evident with cetuximab, we evaluated the antitumor effect of cetuximab on CAL27 and CAL27 miR-223 tumors implanted orthotopically in the mouth floor of nude mice. We confirmed that cetuximab significantly reduced tumor growth of CAL27 tumors. However, when CAL 27 overexpressed miR-223-3p, cetuximab did not significantly inhibit the tumor growth. This result confirmed our observation *in vitro* showing that miR-223-3p promoted tumor resistance to cetuximab. There is no other study examining the impact of miR-223-3p on cetuximab resistance, which represents a critical issue in HNSCC. However, it has previously been reported that miR-223 was able to reverse tumor resistance of EGFR tyrosine kinase inhibitors (TKIs) [42, 43]. For example, Han *et al*. demonstrated that downregulation of miR-223-3p promoted resistance of non-small cell lung cancer cells to erlotinib, an EGFR TKI, through activation of the IGF1R/PI3K/AKT pathway [43]. These conflicting results may be explained by the fact that, in our HNSCC model, pERK2, AKT and pAKT levels increased in CAL27 miR-223 tumors. This miR-223-3p-induced activation of the downstream effectors of the EGFR signaling pathway may explain the resistance to cetuximab observed in our study. The molecular mechanisms leading to this activation occurred downstream of the interaction between EGFR and its ligands since we showed that EGFR expression was not modified by miR-223-3p transfection (Supplementary Figure 1).

In summary, we demonstrated that overexpression of miR-223-3p in the human head and neck cell line resulted in decreased expression of STAT3 but also in decreased formation of neo-vessels in an orthotopic xenograft tumor model. This anti-angiogenic effect has been correlated with high expression areas of miR-223-3p in biopsies from HNSCC patients. Furthermore, using the xenograft tumor model, we demonstrated that miR-223-3p expression impaired the anti-tumoral effect of cetuximab. Since we did not observe necrosis in tumor xenografts (Supplementary Figure 3B), we favor a scenario in which the high neutrophil infiltration allows transfer of miR-223 to malignant cells, which in turn decreases the production of angiogenic factors resulting in hypo vascularization.

Taken together, our results highlight the importance of studying the expression of miR-223-3p level in tumor tissue sections since it may help to predict cetuximab response in patients with HNSCC.

## MATERIALS AND METHODS

### Cell culture

The human head and neck cancer cell line CAL27 was supplied by ATCC (CRL-2095). CAL27 was first transduced with a viral suspension obtained from HEK cells infected with pLenti-Luciferase vector. Bioluminescence from luciferase activity was quantified using an *in vivo* imaging system (IVIS, Caliper LifeSciences) according to the manufacturer’s procedure. CAL27 Luci cells were then infected with lentiviral particles for hsa-miR-223 (Cat. #: PMIRH223PA-1) supplied by System Biosciences following the manufacturer’s instructions. Infection efficiency was measured under a fluorescent microscope one week after the transfection and the GFP positive cells were sorted using a flow cytometer. The sorted cells were used in the experiments.

Cells were cultured at 37°C in controlled atmosphere (5% CO2 and 95% air) with Dulbecco’s Modified Eagles Medium, (Life Technologies) supplemented with 10% heat-inactivated fetal calf serum with penicillin/streptomycin. Prior to injecting the mice, cells were trypsinized and prepared in Ringer lactate solution at 1 × 10^7^ cells/ml. For the proliferation assay, cells were plated at 1 × 10^4^ cells/well in 24-well plates (BD Falcon) at day 0 in growing medium. Cells were detached by trypsin treatment and counted each day. For the wound healing assay, cells were plated at 1 × 10^5^ cells/well in a 6-well plate. At confluency, a wound was made using a yellow tip and the first image was taken (*t* = 0) to measure the distance separating the two rims. At the indicated times, the distance between the two rims was assayed and the percentage of recovery was calculated.

### Patients and samples

Surgically-removed tumors embedded in paraffin-wax blocks from 35 cases of HNSCC were retrieved from the archives of the Laboratory of Clinical and Experimental Pathology (Pasteur Hospital) at the University of Nice, Nice, France. All tumor specimens were collected, stored, and used with the informed consent of the patients (Hospital-Integrated Biobank BB-0033-00025, Pasteur Hospital, Nice, France). The study was approved by the Ethics Committee of the University of Nice-SophiaAntipolis and performed according to the guidelines of the Declaration of Helsinki. The cases, recruited between 2005 and 2010, included squamous cell carcinomas taken exclusively from metastasis-free patients. Cases were included in this study only if a follow-up of at least 5 years was obtained, and clinical data were available. Mean age at surgery was 61 years (range: 39 – 71) and 21 patients were male. Selected patients displayed similar health status and absence of concurrent chronic illnesses, or tumors elsewhere. The primary sites of the carcinomas were: oropharynx (18), hypopharynx (9), and larynx (8). Tumor stage (primary tumor) according to the 2009 American Joint Committee on Cancer staging system was T1 (12), T2 (10,) T3 (8) and T4 (5). Of note, eighty per cent of the carcinomas were keratinizing, with a range of grades: grade I (9), grade II (15), and grade III (11).

### Animal strains and xenograft orthotopic model of HNSCC

This study was approved by the Institutional Care and Use Committee of the University of Nice-Sophia Antipolis. Animal protocols were approved by the committee for Research and Ethics of the PACA region (CIEPAL azur, #PEA 12-153) and followed the European directive 2010/63/UE. NMRI nude mice (nu/nu) were supplied by Janvier laboratories (Le Genest-St-Ile, France). A hundred μl of ringer lactate solution containing 1 × 10^6^ CAL27 Luci +/− miR-223 were injected into the mouth floor of seven-week-old female mice as described previously [44]. On day one, cell implantation was verified by IVIS imaging and groups were stratified (8 to 10 animals per group). For mice receiving treatment, cetuximab (Erbitux, Merck Serono, Darmstadt, Germany) 5 mg/kg, cisplatin (Milan, Amsterdam, Netherlands) and docetaxel (Accord Healthcare, Lille, France) 20 mg/kg were diluted in NaCl 0.9% prior to the injection via the intraperitoneal route (100 μl per injection) at days +3 and +9. Before sacrifice (day 14), luciferase activity was scored.

### Quantitative real time PCR and immunoblotting

Total RNA was isolated from cells using the AllPrep DNA/RNA/Protein Mini Qiagen kit (Qiagen) following the manufacturer’s instruction, and 1 μg was reversed transcribed using the high-capacity cDNA RT kit (Applied Biosystems). RT-qPCR analysis was performed on a StepOne Real-Time PCR system using TaqMan PCR Master Mix (Applied Biosystems; Life Technology), as previously described [45]. Relative changes in gene expression were reported as fold changes compared with the control CAL27 cell line.

Protein extracts were resolved by SDS-PAGE and transferred onto a polyvinylidene difluoride membrane (Immobilon-P; Millipore). After saturation, membranes were incubated overnight with the indicated antibody, washed in Tris-NaCl buffer (TN) supplemented three times with 0.1% Triton X-100 (TNT), and finally with TN buffer. Membranes were then incubated with the secondary anti-rabbit or anti-mouse horseradish peroxidase-conjugated antibody. Bound antibodies were revealed using an ECL system (Pierce) and the signal was quantified with a Pxi Camera (Ozyme, FR). Phospho-ERK (#9101), ERK (#9102), phospho-AKT (#9271), AKT (#9272), Phospho-STAT3 (#9131), STAT3 (#9139) and EGFR (#2085) antibodies were from Cell Signaling and the anti-ACTB (Actin, clone AC40) antibody was from Sigma Aldrich.

### Histology and immunohistochemical analysis

For histopathology, paraffin-embedded tissues were sectioned (4 μm-thick slices), stained with hematoxylin and eosin and evaluated blind by three pathologists (CB, MI, PH).

To investigate the cell-specific distribution of miRNA in normal and head and neck tumors, *in situ* hybridization was performed using 5′- and 3′-end digoxigenin (DIG)-labeled LNA-modified DNA oligonucleotides (LNAs) complementary to the mature miRNA (Exiqon A/S, Denmark). In this study, the global expression of miR-223-3p was examined (LNA-scrambled as a negative control). Scoring of miR-223-3p, neutrophil infiltrate and CD31 expression were done by the pathologists at the invasive front of the tumors.

Immunohistochemical (IHC) staining for KI67, cleaved caspase3, CA9, VEGFR2 and CD31 was performed using tumor sections. Antigen retrieval was performed by boiling sections for 10 min in citrate buffer (pH 6.0) and cooling at RT°, followed by blocking of endogenous peroxidase activity with 0.3% H_2_O_2_ for 30 min. The sections were blocked with 2.5% horse serum in TBS solution for 30 min in a humid chamber prior to incubation with optimal dilutions of anti-Ki67 (Epitomics, 1/300), anti-cleaved-caspase-3 (Imgenex, 1/500), anti-CA9 (Abcam, 1/500), anti-VEGFR2 (Cell Signaling, 1/400), and anti-CD31 (M0823 Dako, clone JC70A, 1/50) overnight at 4°C. Positive cells were detected using an ImmPRESS HRP anti-rabbit detection kit or directly with an anti-Streptavidin-Alexa-594 antibody. The immune complexes were visualized using a Peroxidase Substrate DAB kit (Vector) according to the manufacturer’s protocol, and slides were counterstained with hematoxylin. Blind quantification of brown staining was done as follows: Ki67 and cleaved caspase-3 tumor nuclear staining corresponded to no cells (1), 1 to 10 % (2), 10 to 30% (3), 30 to 50% (4) and > 50% (5). Membranous and cytoplasmic staining of CA9 corresponded to less than 80% (1) or 80 to 100 % of the cells (2). The final score corresponded to a mean of 5 fields per tumor with 5 to 10 tumors per group. Tumor angiogenesis was quantified using both VEGFR2 and CD31 staining. VEGFR2 staining was scored following two criteria: the percentage of positive cells with no cells (1), 1 to 5 % (2), 5 to 30% (3) and > 30 % (4) and the number of aligned positive cells (considered as microvascular density, MVD) with a single cell (1), 2 to 3 cells (2) and more than 4 aligned cells (4). MVD was also measured following the method described by Weidner *at al*. [46]. In brief, scanned slides (NanoZoomer 2.0 HT from Hamamatsu) were initially evaluated at 50× magnification in order to identify and count areas with higher vascular density characterized as hot spots. In addition, 3 representative hot spots measuring 70000 μm^2^ were selected. Images of the selected fields were analyzed at 400× magnification. Any brown endothelial cell clusters (≥ 3 cells) that were clearly separated from adjacent microvessels, tumor cells and other connective tissues were considered as countable microvessels. The total number of vessels counted in each case was divided by the number of hot spots, thus providing the mean MVD for that case.

### Cell survival and clonogenic assay

For cell survival assay, 1 × 10^4^ CAL27 +/− miR-223 cells were seeded on a 96-well plate and, where notified, pre-treated with the indicated dose of cisplatin, cetuximab, docetaxel and fluorouracil (5-FU) for 48 hrs. XTT was added to each well and its cleavage to formazan was followed every 15 minutes over a 10 hr period. Formazan dye was quantified using a scanning multi-well spectrophotometer, as indicated by the manufacturer (Roche, # 11465015001).

For clonogenic assay, 120 CAL27 or CAL27 miR-223 cells were plated on a diameter 60 plate. After 3 weeks, clones were fixed with 4% paraformaldehyde and stained with 0.4% crystal violet. Number and area were quantified using ImageJ software. Where indicated, cells were treated with cetuximab (50 nM) 24 hrs after plating.

### Statistical analysis

All data are represented as mean values and error bars represent SEM. The unpaired *t* test was used to evaluate the statistical significance between groups. The relationship between the presence of neutrophils, the expression of miR-223-3p and the expression of CD31 in controls and tumor samples were analyzed with the Fisher’s exact test.

## SUPPLEMENTARY MATERIALS FIGURES


