# Supplementary material for: MiR-223-3p inhibits angiogenesis and promotes resistance to cetuximab in head and neck squamous cell carcinoma
Source: Oncotarget. 2017 Jul 11;8(34):57174–86. doi: 10.18632/oncotarget.19170 (PMC5593634; doi:10.18632/oncotarget.19170)
Supplement: Supplementary file 1 [file oncotarget-08-57174-s001.pdf]

MiR-223-3p inhibits angiogenesis and promotes resistance to cetuximab in head and neck squamous cell carcinoma

SUPPLEMENTARY MATERIALS

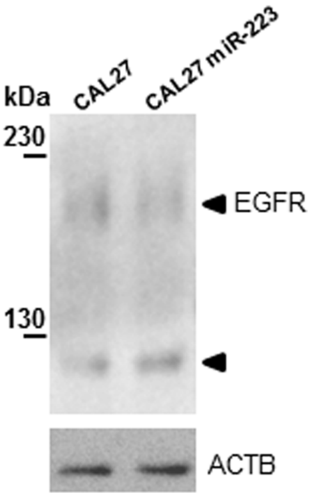

Supplementary Figure 1: Expression of miR-223 does not impact EGFR expression. Immunoblot analysis of EGFR expression by CAL27 and CAL27 miR-223 cells.

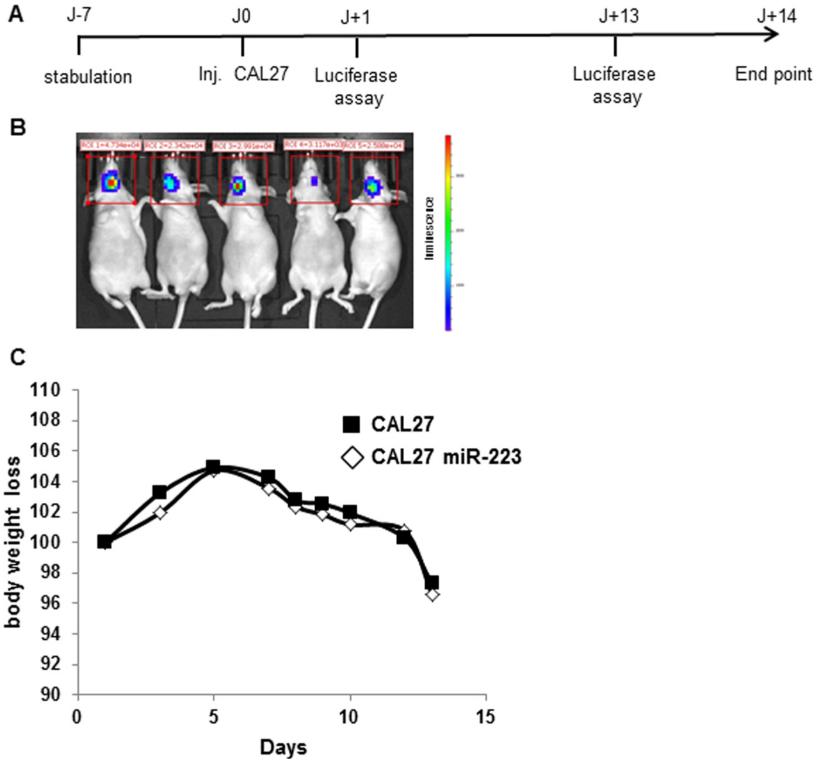

Supplementary Figure 2: Effect of miR-223-3p on orthotopic xenograft. (**A**) *In vivo* experimental schedule. Treatment with cetuximab was done on days 3 and 9. (**B**) Mice groups were made according to their luciferase expression levels. (**C**) Body weight follow-up indicated that mice injected with CAL27 miR-223-3p cells behaved like the control group injected with CAL27 cells.

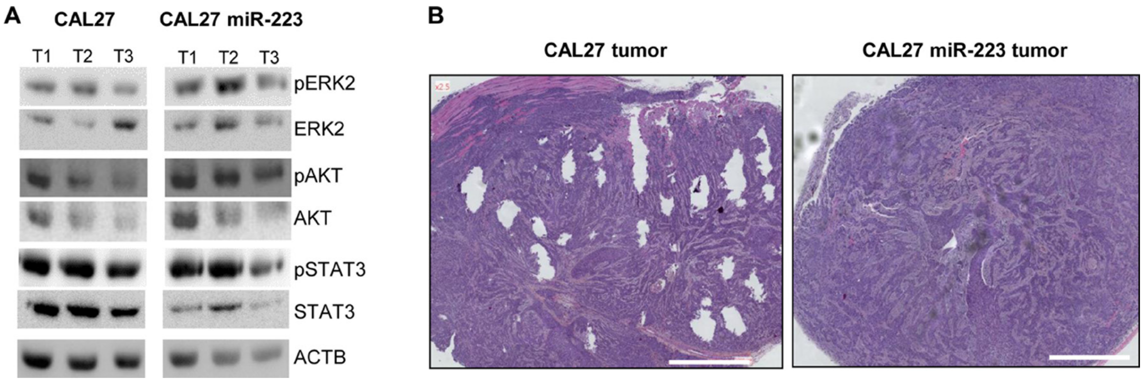

Supplementary Figure 3: (**A**) Immunoblot analysis of ERK2, AKT, STAT3 and their active phosphorylated forms in tumors from CAL27 and CAL27 miR-223 mice. Results showed that STAT3 protein is downregulated in cells expressing miR-223. (**B**) HES staining of tumors obtained after injection of CAL27 and CAL27 miR-223-3p cells highlighting the absence of necrotic areas.

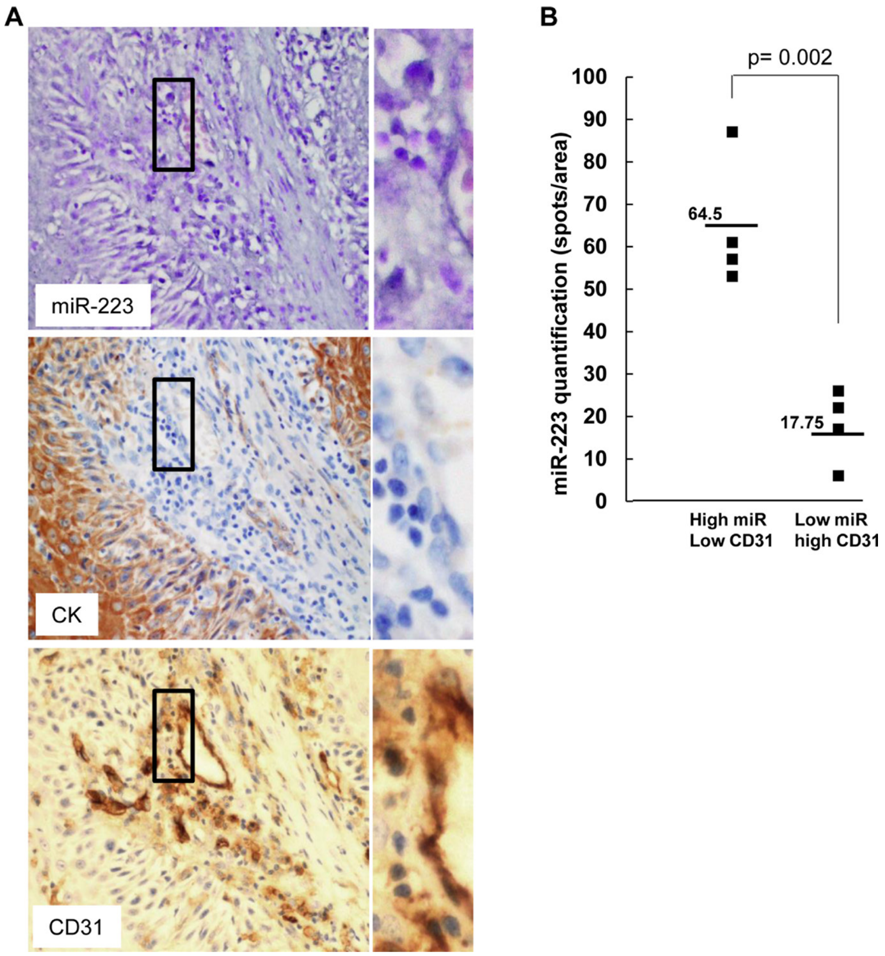

Supplementary Figure 4: Inverse correlation between miR-223-3p presence and CD31 expression. (**A**) Consecutive sections of T2 head and neck tumor stained for miR-223-3p, pan cytokeratin (CK) and CD31 showed that HNSCC tissue sections stained with anti CD31 antibody are negative for both CK and anti-miR-223-3p. This result confirms that miR-223-3p is present in tumor epithelial cells and not expressed in endothelial cells. A representative picture of consecutive sections from 8 tumors stained with miR-223-3p probe and CK is shown (magnification 800×), inset 1600×. (**B**) Quantification of miR-223 expression was done using the Analyze Plug-ins of ImageJ. Five zones per tumor were quantified and 10 areas (36409 pixel2) were counted for each zone. Results are expressed as the number of spots per area (n = 4) and the value of the mean is shown above the bar. Unpaired t test was done for statistical analysis.

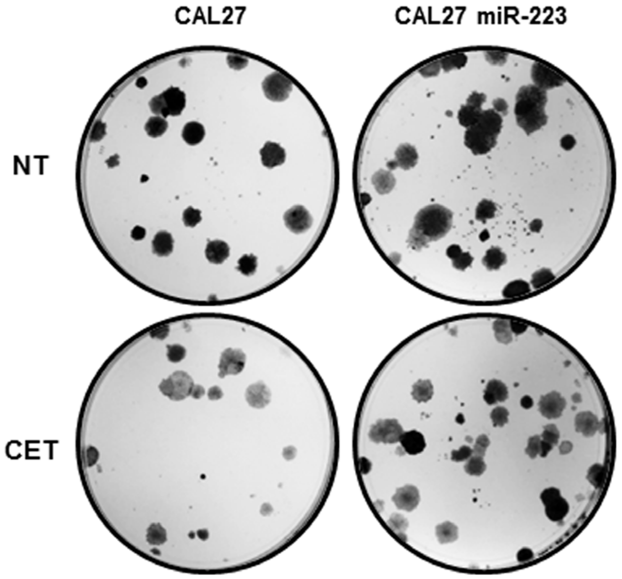

Supplementary Figure 5: Three weeks after plating, treated cells were stained with crystal violet and scanned for quantification, as indicated in the Materials and Methods section.
